# Prevention of lymphocele or seroma after mastectomy and axillary lymphadenectomy for breast cancer: systematic review and meta-analysis

**DOI:** 10.1038/s41598-022-13831-9

**Published:** 2022-06-15

**Authors:** Crestani Adrien, Mahiou Katia, Bodet Marie-Lucile, Roosen Alice, Bonneau Claire, Rouzier Roman

**Affiliations:** 1 Institut Curie, 35, Rue Dailly, 92210 Saint-Cloud, France; 2 Université de Versailles SQY, Université Paris Saclay, UFR des Sciences de la Santé Simone-Veil, Paris, France; 3 Institut Curie, Inserm U900, Saint-Cloud, France

**Keywords:** Breast cancer, Cancer

## Abstract

Seroma or lymphocele remains the most common complication after mastectomy and lymphadenectomy for breast cancer. Many different techniques are available to prevent this complication: wound drainage, reduction of the dead space by flap fixation, use of various types of energy, external compression dressings, shoulder immobilization or physical activity, as well as numerous drugs and glues. We searched MEDLINE, clinicaltrials.gov, Cochrane Library, and Web of Science databases for publications addressing the issue of prevention of lymphocele or seroma after mastectomy and axillary lymphadenectomy. Quality was assessed using Hawker’s quality assessment tool. Incidence of seroma or lymphocele were collected. Fifteen randomized controlled trials including a total of 1766 patients undergoing radical mastectomy and axillary lymphadenectomy for breast cancer were retrieved. The incidence of lymphocele or seroma in the study population was 24.2% (411/1698): 25.2% (232/920) in the test groups and 23.0% (179/778) in the control groups. Neither modification of surgical technique (RR 0.86; 95% CI [0.72, 1.03]) nor application of a medical treatment (RR 0.96; 95% CI [0.72, 1.29]) was effective in preventing lymphocele. On the contrary, decreasing the drainage time increased the risk of lymphocele (RR 1.88; 95% CI [1.43, 2.48). There was no publication bias but the studies were of medium to low quality. To conclude, despite the heterogeneity of study designs, drainage appears to be the most effective technique, although the overall quality of the data is low.

## Introduction

Axillary lymph node dissection (ALND) and mastectomy are performed as part of the surgical management of breast cancer and are associated with significant morbidity, as 70% of patients experience complications^1,2^.

Seromas or lymphoceles are the most common complication of these procedures and can delay local healing and initiation of adjuvant therapy. They are also a source of discomfort for patients. Many techniques have been developed to decrease the risk of seroma formation: wound drainage^3^, reduction of the dead space by flap fixation^4^, use of various types of energy^5^, external compression dressings ^6^, shoulder immobilization or physical activity^7^, as well as numerous drugs and glues^8–11^.

Two previous Cochrane meta-analyses have evaluated fibrin glues and wound drainage and concluded on the inefficacy of fibrin glues and moderate efficiency of drainage supported by low quality studies^3,8^. To our knowledge, no meta-analysis has compared all proposed techniques for seroma prevention after mastectomy and axillary lymphadenectomy.

## Materials and methods

This meta-analysis was performed in accordance with the 2009 Preferred Reporting Items for Systematic Reviews and Meta-analysis (PRISMA) guidelines and the Cochrane Collaboration recommendations^12^. The “Prevention of seroma after breast cancer surgery” trial was registered on the Open Science Framework (OSF) platform https://doi.org/10.17605/OSF.IO/RFVG6.

### Literature search

We searched MEDLINE, clinicaltrials.gov, Cochrane Library, and Web of Science databases for publications of randomized controlled trials (RCT) and clinical trials addressing the issue of prevention of lymphocele or seroma after mastectomy and axillary lymphadenectomy. Various combinations of the following terms were searched: “lymphocele”, “lymphorrhea”, “seroma”, “breast cancer”, “breast surgery”.

### Eligibility criteria

Three authors independently conducted the initial research to evaluate eligibility criteria (AC, MLB, KM). We selected randomized controlled trials and clinical trials published after January 2000 in English, including more than 50 participants, reporting the incidence of lymphocele or seroma after mastectomy and axillary lymphadenectomy for breast cancer. The latest search was performed in March 2021.

The following publications were excluded: retrospective studies, case reports, letters to the editor, publications concerning plastic surgery, brachytherapy or radiation therapy.

### Data collection process and outcome measures

Three authors independently performed data collection using a standardized data extraction table (AC, MLB, KM). The following data were extracted: author, year and country of publication, study characteristics, prevention technique, inclusion and exclusion criteria, number of patients, data necessary to build 2 × 2 contingency tables.

### Statistical analysis

#### Publication bias

A funnel plot was used to visualize publication bias. The estimate of the difference between groups was pooled, depending upon the effect weights of the variance estimate determined in each trial. Egger’s test was used to assed asymmetry of the funnel plot^13^.

#### Outcomes

For dichotomous outcomes, the Mantel–Haenszel method was used for calculation of relative risk (RR) under the fixed-effect and random-effects models^13^. The Forest plot was used for graphic display of the results of the meta-analysis. The heterogeneity of studies was calculated using the I^2^ index. The I^2^ value was interpreted by balancing the direction and magnitude of I^2^ with its statistical significance, using the values in the Cochrane Handbook for Systematic Reviews of Interventions as a guide^14^: 0% to 40%: might not be important; 30% to 60%: may represent moderate heterogeneity; 50% to 90%: may represent substantial heterogeneity; 75% to 100%: represents considerable heterogeneity. Meta‐analyses with insignificant heterogeneity were calculated using the fixed‐effects model^15^. For meta-analyses with low or moderate heterogeneity, the random‐effects model was used^16^. The square around the estimate represents the accuracy of the estimation (sample size) and the horizontal line represents the 95% confidence interval (95% CI).

Data were entered in an Excel file and all statistical analyses were performed using Rstudio software (*RStudio*, *PBC*, *Boston*, *USA*)*.* A P value < 0.05 was considered to be statistically significant.

#### Quality assessment of the studies included

We used a quality assessment tool elaborated by Hawker et al.^17^ in 2002 for systematic review of qualitative evidence. The scale contains nine items assessing abstract/title, introduction/aims, method/data, sampling, data analysis, ethics/bias, results, transferability and implications. Each item is rated as “good”, “fair”, “poor” and “very poor”. Lorenc et al.^18^ added a graduation to this scale by assigning answers from 1 point (very poor) to 4 points (good), to provide a final score for each study (9 to 36 points). The overall quality grades were defined by the following description: grade A (high quality) 30–36 points; grade B (medium quality), 24–29 points and grade C (low quality), 9–24 points. Each of the three readers assessed the studies independently. When differences were observed, a majority agreement was reached.

## Results

### Study selection

**Figure 1:**
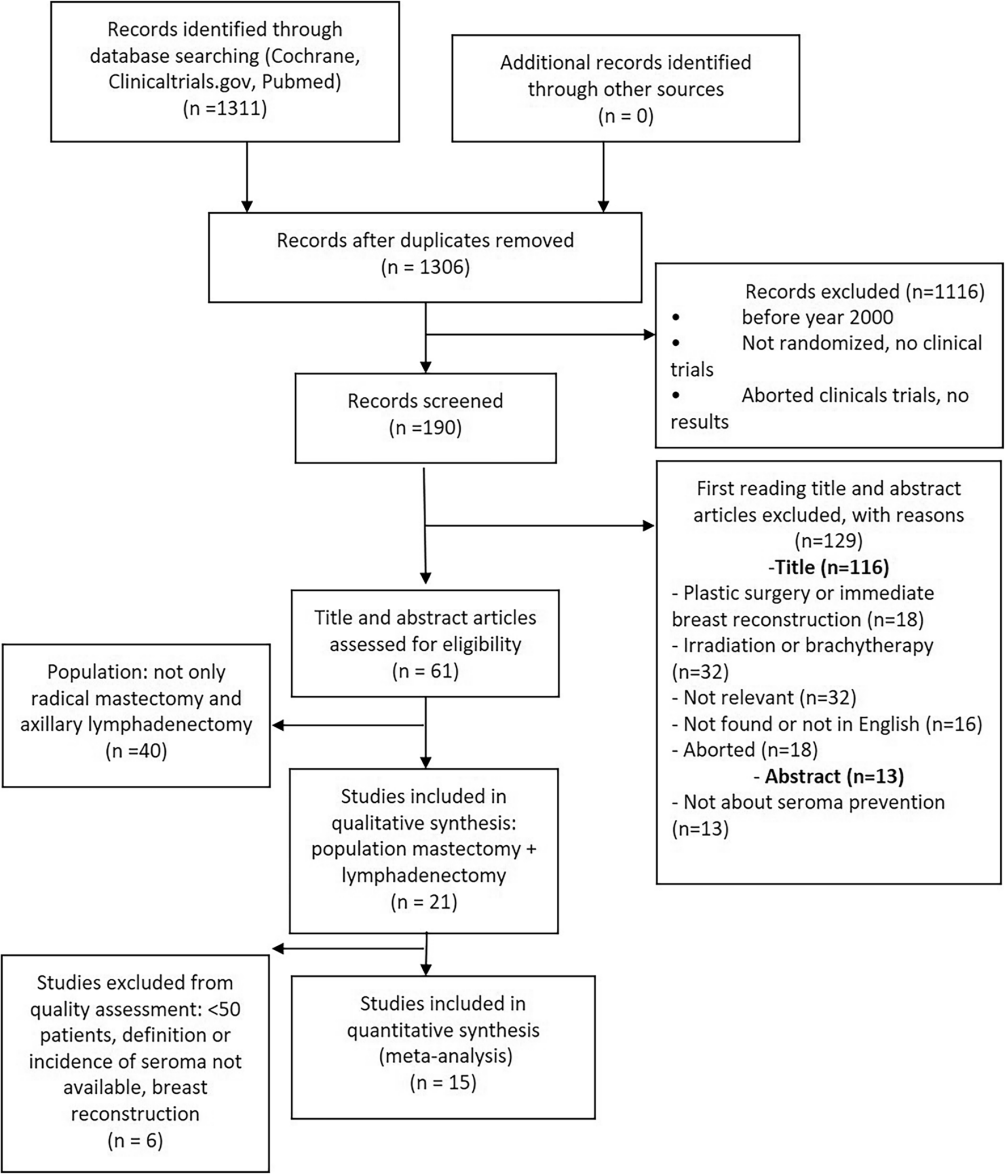
Preferred reporting items for systematic reviews and meta‐analyses (PRISMA) flow diagram of literature screening and selection.

The PRISMA flow diagram explaining the literature search strategy and trial selection is presented in Fig. 1. Fifteen randomized controlled trials including a total of 1766 patients undergoing mastectomy and axillary lymphadenectomy for breast cancer were retrieved from the electronic databases. Analysis was based on 920 patients in the test groups and 878 patients in control groups. The characteristics of the trials included in this meta-analysis are provided in Table 1. The technique used in each article is described in Table 1. The incidence of lymphocele or seroma in the study population was 24.9% (411/1648): 29.5% (271/920) in the test groups and 23.9% (210/878) in the control groups.

**Table 1:** Characteristics of included stories.

Authors	Year	Technique used	Term used	Seroma definition	Number of patients	Incidence of seroma, study population n/N (%)	Incidence of seroma: test group n/N (%)	Incidence of seroma: control group n/N (%)	p-value
Rice et al.^19^	2000	Drug	Seroma	0	62	23/62 (37)	16/30 (53)	7/32 (22)	**0.01**
Gupta et al.^20^	2001	Drain	Lymphocele	Palpation	121	47/121 (38)	31/64 (48)	16/57 (28)	**0.026**
Ali Naki Ulusoy et al.^21^	2003	Glue	Seroma	0	54	8/54 (15)	5/27 (18)	3/27 (11)	> 0.05
Dalberg et al.^22^	2004	Drain	Seroma	Palpation	247	70/247 (28)	48/99 (48)	22/99 (22)	**< 0.001**
		Surgery					39/98 (40)	31/100 (31)	0.2
Chintamani et al.^23^	2005	Drain	Seroma	0	85	3/85 (4)	2/50 (4)	1/35 (3)	> 0.05
Clegg-Lamptey et al.^24^	2007	Drain	Seroma	Palpation	87	33/87 (38)	21/45 (47)	12/42 (29)	0.2
Yiping Gong et al.^25^	2010	Surgery	Seroma	Palpation	201	16/201 (8)	14/101 (14)	2/100 (2)	**< 0.01**
Cabaluna et al.^26^	2013	Drug	Seroma	0	254	35/148 (24)	18/74 (24)	17/74 (23)	0.86
Ribeiro et al.^27^	2013	Surgery	Seroma	0	94	21/94 (22)	8/49 (16)	13/46 (28)	0.16
Khan S et al.^28^	2014	Surgery	Seroma	Palpation	150	41/150 (27)	16/75 (21)	25/75 (33)	0.07
Maia Freire de Oliveira et al.^29^	2014	Physical activity	Seroma	Palpation	96	33/84 (39)	19/43 (44)	14/41 (34)	0.35
Garza-Gangemi et al.^30^	2015	Drug	Seroma	Palpation	80	17/80 (21)	10/50 (20)	7/30 (23)	0.7
Chereau et al.^31^	2016	Drug	Lymphocele	Palpation and needle aspiration volume	90	42/90 (47)	16/42 (38)	26/48 (54)	> 0.05
Kong et al.^32^	2016	Drug	Seroma	0	80	14/80 (18)	2/40 (5)	12/40 (30)	**< 0.01**
Khan M et al.^33^	2017	Drug	Seroma	Palpation and needle aspiration volume and ultrasound	65	8/65 (12)	6/33 (19)	2/32 (6)	> 0.05

Significant values are in bold.

As the study by Dalberg et al.^22^ compared two different techniques in two separate groups of patients, we decided to divide this study into one group treated by drainage and the other group treated by the fascia preservation surgical technique.

### Study characteristics

Study characteristics are described in Table 1. Two of the 15 studies concerned lymphoceles^20,31^, while all of the other studies concerned seromas. Six studies did not specify their definition of seroma, 9 studies reported a clinical definition of seroma or lymphocele (palpation, clinical examination, needle aspiration) and one study used an ultrasound definition. Five studies reported statistically significant results^19,20,22,25,32^.

### Publication bias

The funnel plot did not show any asymmetry (Supplemental Fig. 1). Egger’s test did not reveal any publication bias (p = 0.36).

### Prevention of seroma regardless of the technique

**Figure 2:**
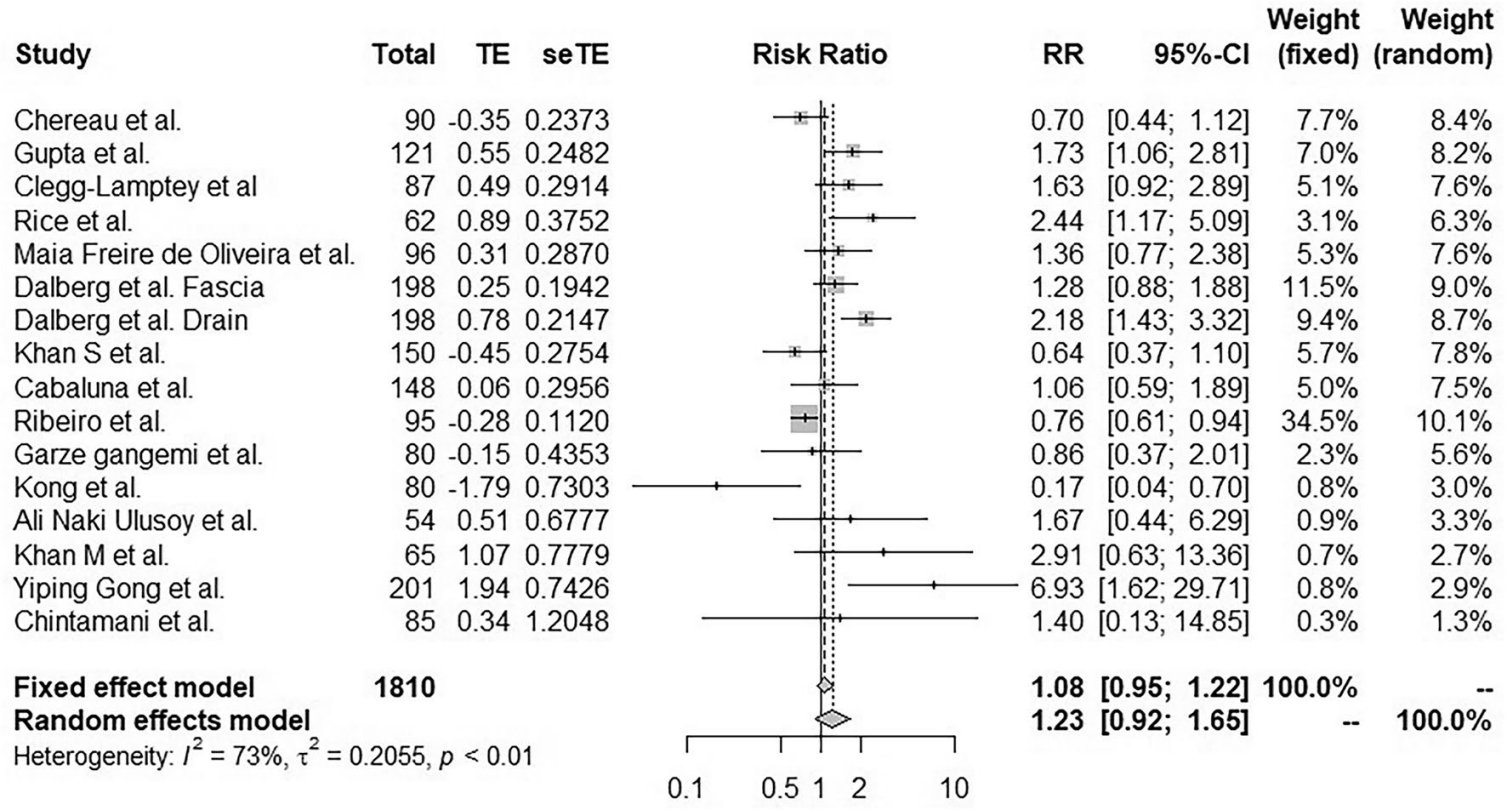
Forest plot for seroma incidence following application of a treatment designed to prevent lymphocele after mastectomy with axillary lymphadenectomy. Risk ratios are shown with 95% confidence intervals.

Significant heterogeneity was observed between the 15 studies (I^2^ = 73%, p < 0.01). Therefore, in the random effects model, none of the techniques allowed statistically significant prevention of lymphocele or seroma formation (RR 1.23; 95% CI [0.92, 1.65]; Fig. 2).

### Prevention of seroma according to the various techniques

#### Medical treatment

**Figure 3:**
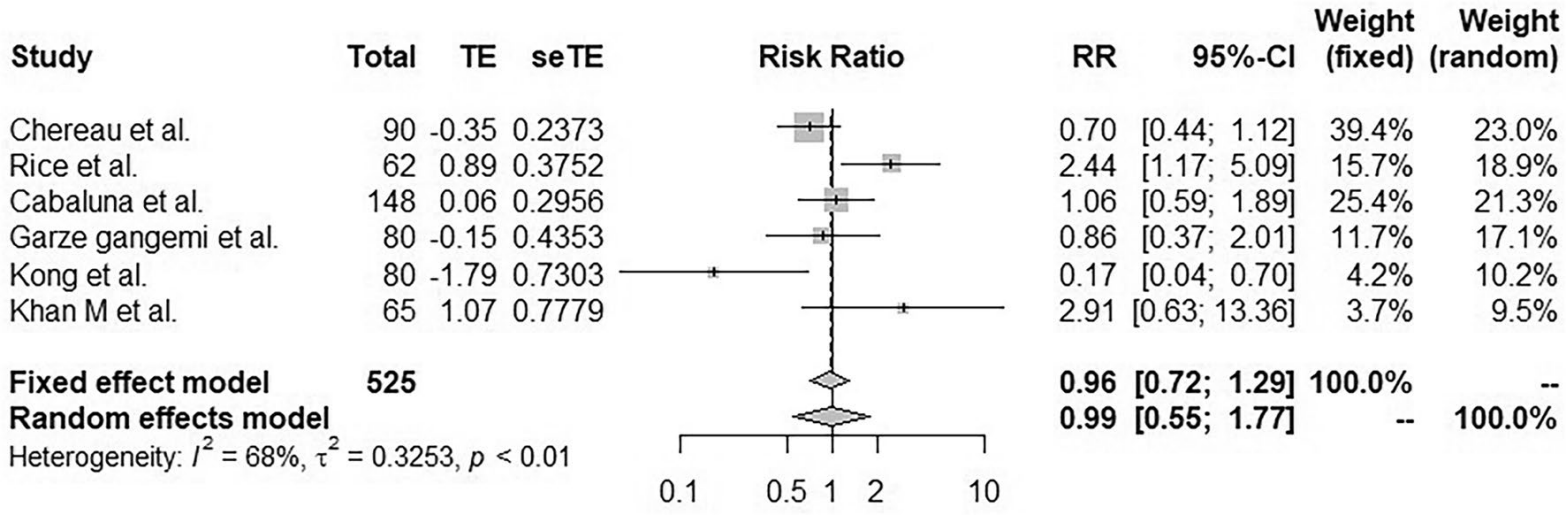
Forest plot for seroma incidence following application of a medical treatment designed to preventing lymphocele after mastectomy with axillary lymphadenectomy. Risk ratios are shown with 95% confidence intervals.

Significant heterogeneity was observed between the 6 studies (I^2^ = 68%, p < 0.01)^19,26,30–32^. Therefore, in the random effects model, medical treatments did not allow statistically significant prevention of lymphocele or seroma (RR 0.96; 95% CI [0.72, 1.29]; Fig. 3).

#### Surgical techniques

**Figure 4:**
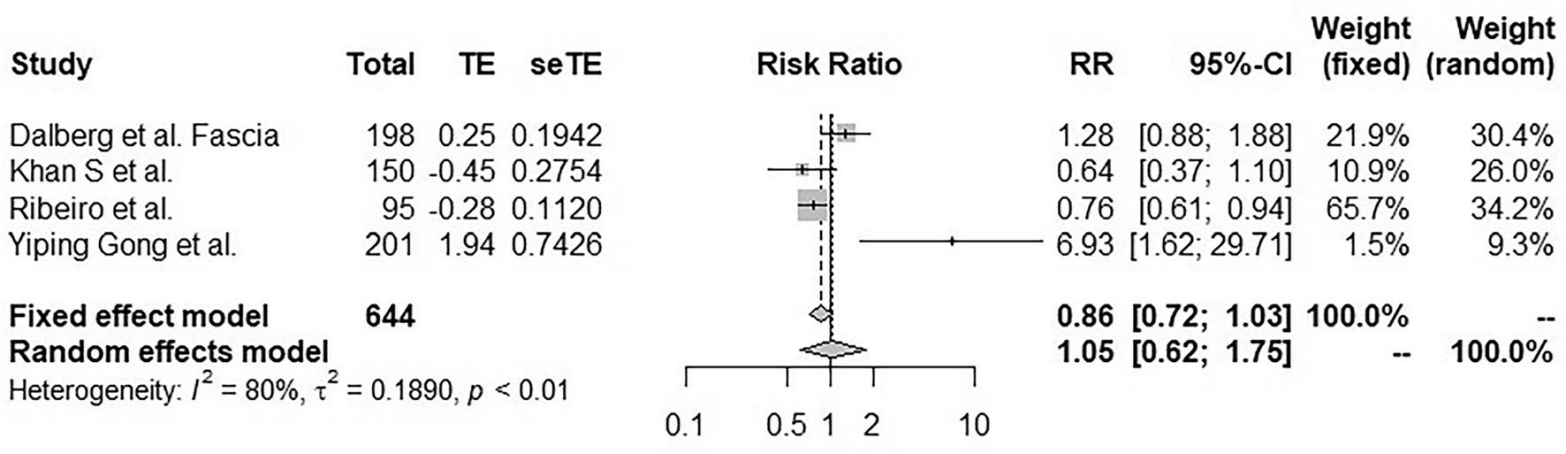
Forest plot for seroma incidence following application of a surgical technique designed to prevent lymphocele after mastectomy with axillary lymphadenectomy. Risk ratios are shown with 95% confidence intervals.

Four studies evaluated surgical techniques for the prevention of lymphocele or seroma. Dalberg et al.^22^ with pectoral fascia preservation, Gong et al.^25^ with lymphatic vessel ligation and padding, Ribeiro^27^ and Khan S et al.^28^ with the use of a harmonic scalpel. Significant heterogeneity was observed between the 4 studies (I^2^ = 77%, p < 0.01)^22,25,27,28^. Therefore, in the random effects model, no specific surgical technique allowed statistically significant prevention of lymphocele or seroma (RR 0.86; 95% CI [0.72, 1.03]; Fig. 4).

#### Modification of the drainage process

**Figure 5:**
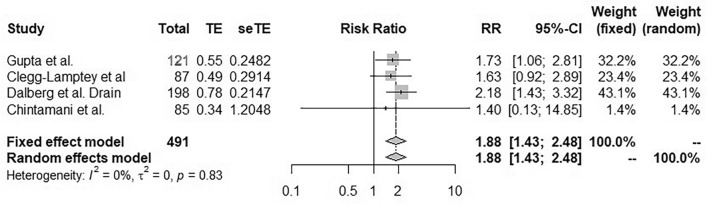
Forest plot for seroma incidence following application of a modified drainage method designed to prevent lymphocele after mastectomy with axillary lymphadenectomy. Risk ratios are shown with 95% confidence intervals.

No heterogeneity was observed between the 4 studies (I^2^ = 0%, p = 0.83)^20,22–24^. Therefore, in the fixed effects model, the risk of lymphocele or seroma was significantly increased by modification of the drainage technique (RR 1.88; 95% CI [1.43, 2.48]; Fig. 5).

#### Other techniques

One study that investigated prevention of lymphocele or seroma using fibrin glue^21^ found this technique to be statistically ineffective (RR 1.36; 95% CI [0.77; 2.38]).

One study that investigated prevention of lymphocele or seroma using physical activity and manual lymphatic drainage^29^ found these technique to be statistically ineffective (RR 1.67; 95% CI [0.44; 6.29]).

### Study quality

The results of the quality assessment are described in Supplemental Table 1. One study was considered to present high quality (Grade A), 8 studies were considered to present medium quality (Grade B), and 6 studies were considered to present low quality (Grade C).

## Discussion

This work represents the first meta-analysis of all techniques proposed for the prevention of lymphocele formation after mastectomy and axillary lymphadenectomy in prospective randomized controlled trials and clinical trials. Global analysis of all of the various techniques showed that they were not effective to prevent lymphocele formation (RR 1.23; 95% CI [0.92, 1.65]). Analysis of studies based on modification of the drainage technique showed a negative effect on seroma prevention (RR 1.88; 95% CI [1.43, 2.48]). Glues and drugs were not effective (RR 1.36; 95% CI [0.77; 2.38], RR 0.96; 95% CI [0.72, 1.29]). The overall quality of these items was moderate with 8 items presenting average quality, 6 items presenting low quality, and only one item presenting high quality.

In this study, we chose to restrict our analysis to the population at high risk of lymphocele or seroma^34^. In our meta-analysis, regardless of the definitions and techniques used to prevent seroma or lymphocele, the overall incidence of these complications was 24.2% (411/1698): 25.2% (232/920) in the test groups and 23.0% (179/778) in the control groups. The reported seroma or lymphocele incidence is dependent on the author’s definition of seroma or lymphocele and the method of detection used. Risk factors for seroma formation include age, body mass index (BMI), tumor size, use of neoadjuvant chemotherapy, type of surgery (MRM versus breast-conserving surgery)^34^, axillary lymph node status, axillary lymph nodes sampled or removed, and subsequently the extent of surgical dead space produced^35^. In our meta-analysis, only one article^21^ considered neoadjuvant chemotherapy to be an exclusion criterion, while most of other studies did not mention neoadjuvant chemotherapy. Other risk factors, except for the type of surgery, were not well documented. This lack of information on risk factors may result in an incidence bias.

The various techniques tested to reduce seroma or lymphocele after breast surgery are based on the different physiological theories. Six studies tested a drug for prevention of seromas. These drugs inhibit the inflammatory or immunopathological response, which is considered to play a role in seroma formation^35^. Four studies evaluated a specific surgical procedure. A French multicenter, superiority, randomized controlled trial, compared seroma formation using quilting suture versus conventional closure with drainage in 320 patients undergoing mastectomy^36^, results have not yet been published. A meta-analysis by Sajid et al. studied application of fibrin glue under skin flaps to prevent seroma-related morbidity following breast and axillary surgery^8^, but this technique failed to reduce the incidence of postoperative seroma (RR 1.02; 95% CI 0.90–1.16, p value = 0.73).

Four studies included in our meta-analysis evaluated modification of the drainage technique. Since 1947 and the first description of drainage after axillary dissection for breast cancer by Murphey^37^, drainage is the technique most commonly used to prevent lymphocele or seroma after radical mastectomy and axillary lymphadenectomy. In 2013, a Cochrane meta-analysis by Thomson et al.^3^ compared wound drainage versus no wound drainage after axillary lymphadenectomy for breast carcinoma. Seven RCTs including 960 participants were identified. The quality of trials was generally low, with several studies at risk of selection bias, and no studies used blinding during treatment or outcome assessment. There was a high level of statistical variation between studies, which therefore reduces the reliability of the evidence. The R for seroma formation was 0.46 ([95% CI 0.23–0.91], p = 0.03) in favor of a reduced incidence of seroma in participants with drains inserted.

Finally, wound drainage appears to be the most effective way to prevent seroma, although no consensus has been reached concerning the optimal duration of drainage. However, persistence of foreign devices under the skin could predispose to surgical site infection. Surgical site infection is one of the possible complications after breast cancer surgery, causing significant morbidity, additional costs and which can delay initiation of adjuvant therapy. In Reiffel’s review^38^ of the potential association between closed-suction drains and surgical site infection, few studies suggested an increased risk of surgical site infection associated with drain placement and no studies attributed a decreased incidence of surgical site infection (including organ/space surgical site infection) with drain placement.

## Conclusions

The lack of consensus concerning the definition of lymphocele or seroma is probably responsible for the heterogeneity of seroma incidence reported in the literature and the inefficacy of the techniques proposed for seroma prevention after breast cancer surgery. However, drainage is the most effective technique currently available. Yet, most studies included in the meta-analysis were evaluated to be of medium or low quality.

## Supplementary Information

Supplementary Figure 1.

Supplementary Table 1.

## Data Availability

The datasets used and/or analysed during the current study available from the corresponding author on reasonable request.

## Abbreviations

ALND: Axillary lymph node dissection
BMI: Body mass index
CI: Confidence interval
MRM: Modified radical mastectomy
OSF: Open science framework
RCT: Randomized control trial
RR: Relative risk

## References

[1] Roses DF, Brooks AD, Harris MN, Shapiro RL, Mitnick J (1999). Complications of level I and II axillary dissection in the treatment of carcinoma of the breast. Ann. Surg..

[2] Lucci A, McCall LM, Beitsch PD, Whitworth PW, Reintgen DS, Blumencranz PW, Leitch AM, Saha S, Hunt KK, Giuliano AE, American College of Surgeons Oncology Group (2007). Surgical complications associated with sentinel lymph node dissection (SLND) plus axillary lymph node dissection compared with SLND alone in the American College of Surgeons Oncology Group Trial Z0011. J. Clin. Oncol..

[3] Thomson DR, Sadideen H, Furniss D (2013). Wound drainage after axillary dissection for carcinoma of the breast. Cochrane Database Syst. Rev..

[4] Granzier RWY, van Bastelaar J, van Kuijk SMJ, Hintzen KFH, Heymans C, Theunissen LLB (2019). Reducing seroma formation and its sequelae after mastectomy by closure of the dead space: The interim analysis of a multi-center, double-blind randomized controlled trial (SAM trial). Breast.

[5] Lumachi F, Basso SMM, Santeufemia DA, Bonamini M, Chiara GB (2013). Ultrasonic dissection system technology in breast cancer: A case-control study in a large cohort of patients requiring axillary dissection. Breast Cancer Res Treat..

[6] O’Hea BJ, Ho MN, Petrek JA (1999). External compression dressing versus standard dressing after axillary lymphadenectomy. Am. J. Surg..

[7] Chen SC, Chen MF (1999). Timing of shoulder exercise after modified radical mastectomy: A prospective study. Chang Yi Xue Za Zhi..

[8] Sajid MS, Hutson KH, Rapisarda IF, Bonomi R (2013). Fibrin glue instillation under skin flaps to prevent seroma-related morbidity following breast and axillary surgery. Cochrane Database Syst. Rev..

[9] Suarez-Kelly LP, Pasley WH, Clayton EJ, Povoski SP, Carson WE, Rudolph R (2019). Effect of topical microporous polysaccharide hemospheres on the duration and amount of fluid drainage following mastectomy: A prospective randomized clinical trial. BMC Cancer.

[10] Gauthier T, Garuchet-Bigot A, Marin B, Mollard J, Loum O, Fermeaux V (2012). Lanreotide Autogel 90 mg and lymphorrhea prevention after axillary node dissection in breast cancer: A phase III double blind, randomized, placebo-controlled trial. Eur. J. Surg. Oncol..

[11] Benevento R, Santoriello A, Pellino G, Sciaudone G, Candilio G, De Fatico GS (2014). The effects of low-thrombin fibrin sealant on wound serous drainage, seroma formation and length of postoperative stay in patients undergoing axillary node dissection for breast cancer: A randomized controlled trial. Int. J. Surg..

[12] Moher D, Liberati A, Tetzlaff J, Altman DG, PRISMA Group (2009). Preferred reporting items for systematic reviews and meta-analyses: The PRISMA statement. PLoS Med..

[13] Egger M, Davey Smith G, Schneider M, Minder C (1997). Bias in meta-analysis detected by a simple, graphical test. BMJ.

[14] Higgins JPT, Altman DG, Gøtzsche PC, Jüni P, Moher D, Oxman AD (2011). The Cochrane collaboration’s tool for assessing risk of bias in randomised trials. BMJ.

[15] Demets DL (1987). Methods for combining randomized clinical trials: strengths and limitations. Stat. Med..

[16] DerSimonian R, Laird N (1986). Meta-analysis in clinical trials. Control Clin. Trials..

[17] Hawker S, Payne S, Kerr C, Hardey M, Powell J (2002). Appraising the evidence: Reviewing disparate data systematically. Qual. Health Res..

[18] Lorenc T, Petticrew M, Whitehead M, Neary D, Clayton S, Wright K (2014). Crime, fear of crime and mental health: Synthesis of theory and systematic reviews of interventions and qualitative evidence. Public Health Res..

[19] Rice DC, Morris SM, Sarr MG, Farnell MB, van Heerden JA, Grant CS (2000). Intraoperative topical tetracycline sclerotherapy following mastectomy: A prospective, randomized trial. J. Surg. Oncol..

[20] Gupta R, Pate K, Varshney S, Goddard J, Royle GT (2001). A comparison of 5-day and 8-day drainage following mastectomy and axillary clearance. Eur. J. Surg. Oncol..

[21] Ulusoy AN, Polat C, Alvur M, Kandemir B, Bulut F (2003). Effect of fibrin glue on lymphatic drainage and on drain removal time after modified radical mastectomy: A prospective randomized study. Breast J..

[22] Dalberg K, Johansson H, Signomklao T, Rutqvist LE, Bergkvist L, Frisell J (2004). A randomised study of axillary drainage and pectoral fascia preservation after mastectomy for breast cancer. Eur. J. Surg. Oncol..

[23] Chintamani A, Singhal V, Singh J, Bansal A, Saxena S (2005). Half versus full vacuum suction drainage after modified radical mastectomy for breast cancer: A prospective randomized clinical trial [ISRCTN24484328]. BMC Cancer.

[24] Clegg-Lamptey JNA, Dakubo JCB, Hodasi WM (2007). Comparison of four-day and ten-day post-mastectomy passive drainage in Accra, Ghana. East Afr. Med. J..

[25] Gong Y, Xu J, Shao J, Cheng H, Wu X, Zhao D (2010). Prevention of seroma formation after mastectomy and axillary dissection by lymph vessel ligation and dead space closure: A randomized trial. Am. J. Surg..

[26] Cabaluna ND, Uy GB, Galicia RM, Cortez SC, Yray MDS, Buckley BS (2013). A randomized, double-blinded placebo-controlled clinical trial of the routine use of preoperative antibiotic prophylaxis in modified radical mastectomy. World J. Surg..

[27] Ribeiro GHFP, Kerr LM, Haikel RL, Peres SV, Matthes AGZ, Depieri Michelli RA (2013). Modified radical mastectomy: A pilot clinical trial comparing the use of conventional electric scalpel and harmonic scalpel. Int. J. Surg..

[28] Khan S, Khan S, Chawla T, Murtaza G (2014). Harmonic scalpel versus electrocautery dissection in modified radical mastectomy: A randomized controlled trial. Ann. Surg. Oncol..

[29] de Oliveira MMF, de Rezende LF, de Amaral MTP, Pinto e Silva MP, Morais SS, Gurgel MSC (2014). Manual lymphatic drainage versus exercise in the early postoperative period for breast cancer. Physiother. Theory Pract..

[30] Garza-Gangemi AM, Barquet-Muñoz SA, Villarreal-Colín SP, Medina-Franco H, Cortés-González R, Vilar-Compte D (2015). Randomized phase II study of talc versus iodopovidone for the prevention of seroma formation following modified radical mastectomy. Rev. Investig. Clin. Organo Hosp. Enfermedades Nutr..

[31] Chéreau E, Uzan C, Boutmy-Deslandes E, Zohar S, Bézu C, Mazouni C (2016). Evaluation of the effects of pasireotide LAR administration on lymphocele prevention after axillary node dissection for breast cancer: Results of a randomized non-comparative phase 2 study. PLoS ONE.

[32] Kong D, Liu Y, Li Z, Cui Q, Wang K, Wu K (2017). OK-432 (sapylin) reduces seroma formation after axillary lymphadenectomy in breast cancer. J. Investig. Surg..

[33] Khan MA (2017). Effect of preoperative intravenous steroids on seroma formation after modified radical mastectomy. J. Ayub. Med. Coll. Abbottabad..

[34] Kuroi K, Shimozuma K, Taguchi T, Imai H, Yamashiro H, Ohsumi S (2006). Evidence-based risk factors for seroma formation in breast surgery. Jpn. J. Clin. Oncol..

[35] McCaul JA, Aslaam A, Spooner RJ, Louden I, Cavanagh T, Purushotham AD (2000). Aetiology of seroma formation in patients undergoing surgery for breast cancer. Breast.

[36] Ouldamer L, Bonastre J, Brunet-Houdard S, Body G, Giraudeau B, Caille A (2016). Dead space closure with quilting suture versus conventional closure with drainage for the prevention of seroma after mastectomy for breast cancer (QUISERMAS): Protocol for a multicentre randomised controlled trial. BMJ Open.

[37] Murphey DR (1947). The use of atmospheric pressure in obliterating axillary dead space following radical mastectomy. South Surg..

[38] Reiffel AJ, Barie PS, Spector JA (2013). A multi-disciplinary review of the potential association between closed-suction drains and surgical site infection. Surg. Infect..

