# Supplementary figures and images for: Prevention of lymphocele or seroma after mastectomy and axillary lymphadenectomy for breast cancer: systematic review and meta-analysis
Source: Sci Rep. 2022 Jun 15;12:10016. doi: 10.1038/s41598-022-13831-9 (PMC9200791; doi:10.1038/s41598-022-13831-9)

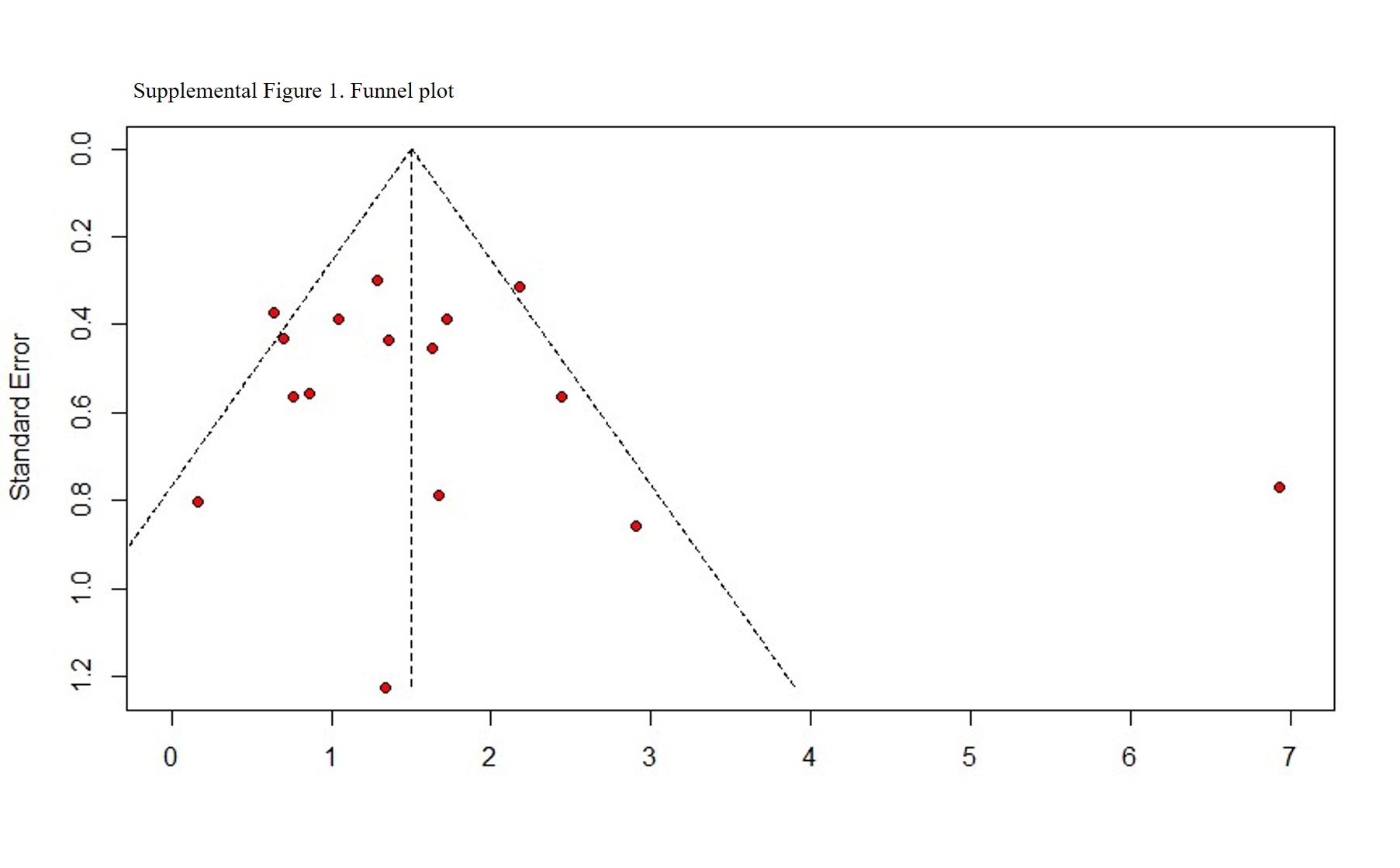

Supplement: Supplementary file 1 — Supplementary Figure 1. [file 41598_2022_13831_MOESM1_ESM.jpg]
