# Supplementary material for: Prevention of lymphocele or seroma after mastectomy and axillary lymphadenectomy for breast cancer: systematic review and meta-analysis
Source: Sci Rep. 2022 Jun 15;12:10016. doi: 10.1038/s41598-022-13831-9 (PMC9200791; doi:10.1038/s41598-022-13831-9)
Supplement: Supplementary file 2 — Supplementary Table 1. [file 41598_2022_13831_MOESM2_ESM.docx]

| Authors | Abstract/Title | Introduction/  Aims | Method/  Data | Sampling | Data analysis | Ethics/  Bias | Results | Transferability | Implications | Quality |
| --- | --- | --- | --- | --- | --- | --- | --- | --- | --- | --- |
| Rice *et al*. (26) | Fair | Poor | Fair | Fair | Fair | Fair | Fair | Poor | Poor | 24 |
| Gupta *et al*. (20) | Good | Fair | Fair | Poor | Fair | Poor | Fair | Fair | Fair | 26 |
| Ali Naki Ulusoy *et al*. (32) | Fair | Fair | Poor | Poor | Very poor | Very poor | Fair | Poor | Poor | 19 |
| Dalberg *et al*. (19) | Good | Good | Good | Fair | Fair | Poor | Fair | Poor | Fair | 28 |
| Chintamani *et al*. (37) | Good | Poor | Fair | Very poor | Poor | Very poor | Poor | Poor | Poor | 19 |
| Clegg-Lamptey *et al.* (38) | Good | Good | Fair | Poor | Poor | Very poor | Poor | Poor | Very poor | 21 |
| Yiping Gong *et al*. (23) | Fair | Fair | Poor | Poor | Poor | Poor | Fair | Poor | Poor | 21 |
| Cabaluna *et al*. (27) | Good | Good | Fair | Poor | Fair | Poor | Fair | Poor | Poor | 25 |
| Ribeiro *et al*. (30) | Good | Fair | Poor | Poor | Fair | Fair | Fair | Poor | Poor | 24 |
| Khan S *et al*. (31) | Good | Fair | Poor | Fair | Fair | Poor | Fair | Fair | Good | 27 |
| Maia Freire de Oliveira *et al*. (39) | Fair | Poor | Fair | Good | Good | Good | Fair | Fair | Poor | 28 |
| Garza-Gangemi *et al*. (28) | Fair | Good | Poor | Poor | Fair | Fair | Fair | Poor | Very poor | 23 |
| Chereau *et al*. (21) | Good | Good | Fair | Fair | Good | Good | Good | Good | Good | 34 |
| Kong *et al*.  (22) | Fair | Fair | Fair | Good | Fair | Fair | Fair | Fair | Poor | 27 |
| Khan M *et al*.  (29) | Fair | Poor | Poor | Very poor | Poor | Very poor | Poor | Poor | Very poor | 16 |

Supplemental table 1: Quality assessment of included studies.
